# Supplementary material for: Modeling glioblastoma heterogeneity as a dynamic network of cell states
Source: Mol Syst Biol. 2021 Sep 16;17(9):e10105. doi: 10.15252/msb.202010105 (PMC8444284; doi:10.15252/msb.202010105)
Supplement: Supplementary file 5 — Source Data for Figure 3 [file MSB-17-e10105-s001.zip › Figure3A_sourcedata/GSEA_3065/hallmarks_state2.GseaPreranked.1623416269049/HALLMARK_APICAL_JUNCTION.html]

Details for gene set HALLMARK\_APICAL\_JUNCTION[GSEA]

|  || Dataset | state2 |
| Phenotype | NoPhenotypeAvailable |
| Upregulated in class | na\_neg |
| GeneSet | HALLMARK\_APICAL\_JUNCTION |
| Enrichment Score (ES) | -0.4040928 |
| Normalized Enrichment Score (NES) | -1.5944558 |
| Nominal p-value | 0.00877193 |
| FDR q-value | 0.011862042 |
| FWER p-Value | 0.081 |
Table: GSEA Results Summary

  

Fig 1: Enrichment plot: HALLMARK\_APICAL\_JUNCTION      
 Profile of the Running ES Score & Positions of GeneSet Members on the Rank Ordered List

  

| PROBE | GENE SYMBOL | GENE\_TITLE | RANK IN GENE LIST | RANK METRIC SCORE | RUNNING ES | CORE ENRICHMENT || 1 | YWHAH |  |  | 221 | 0.271 | 0.0075 | No |
| 2 | TUBG1 |  |  | 262 | 0.246 | 0.0315 | No |
| 3 | PFN1 |  |  | 352 | 0.216 | 0.0469 | No |
| 4 | MYL12B |  |  | 383 | 0.208 | 0.0677 | No |
| 5 | ACTB |  |  | 571 | 0.168 | 0.0670 | No |
| 6 | MAP3K20 |  |  | 678 | 0.152 | 0.0732 | No |
| 7 | HADH |  |  | 778 | 0.141 | 0.0788 | No |
| 8 | GAMT |  |  | 825 | 0.135 | 0.0895 | No |
| 9 | ARPC2 |  |  | 1033 | 0.117 | 0.0808 | No |
| 10 | EPB41L2 |  |  | 1200 | 0.105 | 0.0751 | No |
| 11 | WASL |  |  | 1207 | 0.104 | 0.0864 | No |
| 12 | ZYX |  |  | 1511 | 0.086 | 0.0638 | No |
| 13 | HRAS |  |  | 1748 | 0.075 | 0.0471 | No |
| 14 | GTF2F1 |  |  | 1785 | 0.073 | 0.0516 | No |
| 15 | TJP1 |  |  | 1811 | 0.072 | 0.0573 | No |
| 16 | TIAL1 |  |  | 2192 | 0.058 | 0.0232 | No |
| 17 | ACTG1 |  |  | 2364 | 0.052 | 0.0108 | No |
| 18 | SORBS3 |  |  | 2390 | 0.051 | 0.0140 | No |
| 19 | CAP1 |  |  | 2568 | 0.045 | 0.0002 | No |
| 20 | CRAT |  |  | 2856 | 0.038 | -0.0262 | No |
| 21 | EVL |  |  | 2873 | 0.037 | -0.0236 | No |
| 22 | FSCN1 |  |  | 2952 | 0.035 | -0.0280 | No |
| 23 | MYH10 |  |  | 3132 | 0.031 | -0.0436 | No |
| 24 | LIMA1 |  |  | 3279 | 0.028 | -0.0560 | No |
| 25 | PARVA |  |  | 3378 | 0.026 | -0.0635 | No |
| 26 | IKBKG |  |  | 3506 | 0.024 | -0.0744 | No |
| 27 | MVD |  |  | 3516 | 0.024 | -0.0726 | No |
| 28 | TSPAN4 |  |  | 3929 | 0.017 | -0.1149 | No |
| 29 | GNAI2 |  |  | 4037 | 0.015 | -0.1246 | No |
| 30 | NF2 |  |  | 4275 | 0.011 | -0.1488 | No |
| 31 | COL16A1 |  |  | 4589 | 0.006 | -0.1817 | No |
| 32 | PBX2 |  |  | 4670 | 0.005 | -0.1898 | No |
| 33 | DHX16 |  |  | 4801 | 0.003 | -0.2034 | No |
| 34 | VAV2 |  |  | 4836 | 0.002 | -0.2068 | No |
| 35 | MAPK11 |  |  | 4920 | 0.001 | -0.2156 | No |
| 36 | PARD6G |  |  | 4938 | 0.001 | -0.2173 | No |
| 37 | RSU1 |  |  | 5056 | -0.001 | -0.2298 | No |
| 38 | NECTIN1 |  |  | 5211 | -0.003 | -0.2459 | No |
| 39 | TMEM8B |  |  | 5293 | -0.005 | -0.2540 | No |
| 40 | MAP4K2 |  |  | 5401 | -0.007 | -0.2647 | No |
| 41 | GNAI1 |  |  | 5503 | -0.009 | -0.2746 | No |
| 42 | SKAP2 |  |  | 5558 | -0.009 | -0.2793 | No |
| 43 | SIRPA |  |  | 5641 | -0.011 | -0.2869 | No |
| 44 | MSN |  |  | 5667 | -0.011 | -0.2883 | No |
| 45 | CADM3 |  |  | 5686 | -0.011 | -0.2889 | No |
| 46 | MPP5 |  |  | 5773 | -0.013 | -0.2967 | No |
| 47 | AKT3 |  |  | 5783 | -0.013 | -0.2962 | No |
| 48 | INSIG1 |  |  | 5815 | -0.013 | -0.2979 | No |
| 49 | VASP |  |  | 5834 | -0.014 | -0.2983 | No |
| 50 | PTK2 |  |  | 5931 | -0.015 | -0.3068 | No |
| 51 | INPPL1 |  |  | 6059 | -0.017 | -0.3184 | No |
| 52 | PIK3CB |  |  | 6087 | -0.018 | -0.3192 | No |
| 53 | AKT2 |  |  | 6104 | -0.018 | -0.3189 | No |
| 54 | TAOK2 |  |  | 6113 | -0.018 | -0.3176 | No |
| 55 | ACTN4 |  |  | 6190 | -0.020 | -0.3235 | No |
| 56 | ADAM23 |  |  | 6221 | -0.020 | -0.3244 | No |
| 57 | MAPK14 |  |  | 6426 | -0.024 | -0.3435 | No |
| 58 | NLGN3 |  |  | 6477 | -0.025 | -0.3460 | No |
| 59 | PLCG1 |  |  | 6504 | -0.025 | -0.3458 | No |
| 60 | SHROOM2 |  |  | 6628 | -0.028 | -0.3558 | No |
| 61 | STX4 |  |  | 6663 | -0.029 | -0.3562 | No |
| 62 | NLGN2 |  |  | 6720 | -0.030 | -0.3587 | No |
| 63 | RASA1 |  |  | 6745 | -0.030 | -0.3578 | No |
| 64 | MDK |  |  | 6783 | -0.031 | -0.3582 | No |
| 65 | KCNH2 |  |  | 6934 | -0.035 | -0.3702 | No |
| 66 | SYMPK |  |  | 6937 | -0.035 | -0.3664 | No |
| 67 | CLDN15 |  |  | 6996 | -0.036 | -0.3685 | No |
| 68 | CTNND1 |  |  | 6998 | -0.036 | -0.3644 | No |
| 69 | ADAM15 |  |  | 7007 | -0.037 | -0.3610 | No |
| 70 | NEXN |  |  | 7138 | -0.040 | -0.3703 | No |
| 71 | BAIAP2 |  |  | 7169 | -0.041 | -0.3688 | No |
| 72 | CTNNA1 |  |  | 7279 | -0.044 | -0.3754 | No |
| 73 | SPEG |  |  | 7350 | -0.046 | -0.3776 | No |
| 74 | ACTN1 |  |  | 7366 | -0.046 | -0.3738 | No |
| 75 | CX3CL1 |  |  | 7596 | -0.053 | -0.3923 | No |
| 76 | CNN2 |  |  | 7676 | -0.055 | -0.3943 | No |
| 77 | RRAS |  |  | 7768 | -0.059 | -0.3973 | Yes |
| 78 | PKD1 |  |  | 7772 | -0.059 | -0.3909 | Yes |
| 79 | CNTN1 |  |  | 7792 | -0.059 | -0.3861 | Yes |
| 80 | AMH |  |  | 7894 | -0.063 | -0.3897 | Yes |
| 81 | ARHGEF6 |  |  | 7988 | -0.067 | -0.3919 | Yes |
| 82 | LAYN |  |  | 8032 | -0.068 | -0.3887 | Yes |
| 83 | SDC3 |  |  | 8064 | -0.070 | -0.3840 | Yes |
| 84 | BMP1 |  |  | 8090 | -0.071 | -0.3785 | Yes |
| 85 | EGFR |  |  | 8094 | -0.071 | -0.3706 | Yes |
| 86 | FBN1 |  |  | 8107 | -0.072 | -0.3637 | Yes |
| 87 | TSC1 |  |  | 8114 | -0.072 | -0.3560 | Yes |
| 88 | NFASC |  |  | 8136 | -0.074 | -0.3498 | Yes |
| 89 | THY1 |  |  | 8139 | -0.074 | -0.3415 | Yes |
| 90 | CDH4 |  |  | 8177 | -0.076 | -0.3366 | Yes |
| 91 | PTEN |  |  | 8202 | -0.077 | -0.3303 | Yes |
| 92 | NECTIN3 |  |  | 8222 | -0.078 | -0.3233 | Yes |
| 93 | EXOC4 |  |  | 8300 | -0.083 | -0.3220 | Yes |
| 94 | ITGB4 |  |  | 8365 | -0.086 | -0.3189 | Yes |
| 95 | SHC1 |  |  | 8385 | -0.088 | -0.3108 | Yes |
| 96 | NECTIN2 |  |  | 8580 | -0.102 | -0.3199 | Yes |
| 97 | CERCAM |  |  | 8593 | -0.102 | -0.3094 | Yes |
| 98 | CADM2 |  |  | 8653 | -0.107 | -0.3033 | Yes |
| 99 | NF1 |  |  | 8661 | -0.108 | -0.2916 | Yes |
| 100 | MPZL1 |  |  | 8701 | -0.111 | -0.2830 | Yes |
| 101 | TGFBI |  |  | 8761 | -0.117 | -0.2758 | Yes |
| 102 | MYH9 |  |  | 8778 | -0.119 | -0.2638 | Yes |
| 103 | JAM3 |  |  | 8782 | -0.120 | -0.2503 | Yes |
| 104 | CD276 |  |  | 8831 | -0.127 | -0.2408 | Yes |
| 105 | PIK3R3 |  |  | 8840 | -0.127 | -0.2270 | Yes |
| 106 | DLG1 |  |  | 8857 | -0.130 | -0.2137 | Yes |
| 107 | B4GALT1 |  |  | 8942 | -0.140 | -0.2066 | Yes |
| 108 | SGCE |  |  | 8959 | -0.143 | -0.1919 | Yes |
| 109 | CDH11 |  |  | 8983 | -0.146 | -0.1775 | Yes |
| 110 | CD99 |  |  | 9224 | -0.203 | -0.1799 | Yes |
| 111 | ITGA3 |  |  | 9226 | -0.203 | -0.1566 | Yes |
| 112 | ADAM9 |  |  | 9285 | -0.226 | -0.1367 | Yes |
| 113 | MMP2 |  |  | 9307 | -0.235 | -0.1119 | Yes |
| 114 | ITGB1 |  |  | 9388 | -0.339 | -0.0813 | Yes |
| 115 | VCAN |  |  | 9396 | -0.364 | -0.0402 | Yes |
| 116 | VCL |  |  | 9405 | -0.389 | 0.0038 | Yes |
Table: GSEA details [plain text format]

  

Fig 2: HALLMARK\_APICAL\_JUNCTION: Random ES distribution      
 Gene set null distribution of ES for **HALLMARK\_APICAL\_JUNCTION**
